# A292 ACUTE PANCREATITIS AS THE FIRST PRESENTATION OF GRANULOMATOSIS WITH POLYANGIITIS (GPA): A CASE REPORT

**DOI:** 10.1093/jcag/gwac036.292

**Published:** 2023-03-07

**Authors:** M Youssef, M Sedarous, L Hookey

**Affiliations:** 1 Internal Medicine, University of Toronto, Toronto; 2 Gastroenterology, Queen's University, Kingston, Canada

## Abstract

**Background:**

Granulomatosis with Polyangiitis (GPA) is a rare necrotizing ANCA-associated vasculitis characterized by inflammation in small-sized arteries. GPA often presents with a triad of a) upper (nasal obstruction, sinusitis, crusting rhinitis) and lower respiratory tract (lung nodules, alveolar hemorrhage) b) systemic vasculitis and c) kidney involvement (necrotizing glomerulonephritis). However, gastrointestinal involvement is exceedingly rare and only occurs in about 5-11% of GPA cases. Specifically, recurrent acute pancreatitis is even more uncommon.

**Purpose:**

To raise awareness about systemic vasculitides in cases of idiopathic acute pancreatitis

**Method:**

A 48-year-old female was seen in an outpatient gastroenterology clinic for recurrent idiopathic pancreatitis. She reported a six-month history of intermittent sharp epigastric pain associated with a rise in lipase. The patient noted having episodes of acute sinusitis shortly prior to the onset of her epigastric pain. Her initial labwork revealed normal creatinine and liver enzymes, elevated total bilirubin 24, lipase 316 and mildly high CRP 16.1 and ESR 27 which normalized on repeat blood work. Abdominal US was unremarkable with no gallstones, intra- or extrahepatic duct dilatation. An abdominal MRI revealed segmental enlargement of the distal tail/body of the pancreas consistent with resolving focal pancreatitis. A follow-up MRI 5 weeks later revealed progression of pancreatic swelling and intermittent narrowing of the pancreatic duct. These findings were suspicious for an inflammatory process such as autoimmune pancreatitis. The patient then underwent EUS-guided examination and FNA of her pancreas. Pathology revealed focal chronic pancreatitis with fibrosis and mild lymphocytic infiltrate, but was not suggestive of autoimmune pancreatitis. She was then treated empirically with a 3-month course of prednisone and had significant improvement in her symptoms and interval resolution of pancreatic inflammation on repeat MRI. After discontinuation of her steroids, her symptoms recurred with intermittent epigastric pain, facial pain and sinusitis. She was then seen by rheumatology and an autoimmune panel was ordered.

**Result(s):**

Autoimmune work-up revelead positive anti-PR3 (27 RU/ml), negative anti-MPO and normal IgG-4 levels. Ultimately a diagnosis of limited GPA, which spares the kidneys, was made given the patient’s clinical presentation and positive anti-PR3 antibody. Since there was no life-threatening organ involvement, she was elected to start methotrexate therapy and had significant improvement in her symptoms. The plan is to stay on methotrexate for at least 24 months and have regular follow-up to ensure clinical stability.

**Conclusion(s):**

Acute pancreatitis is a rare initial presentation of GPA vasculitis. It is important to consider autoimmune disease and systemic vasculitides in cases of idiopathic pancreatitis after ruling out common causes. Early diagnosis and therapy allow for high rates of remission and improved survival rates.

**Please acknowledge all funding agencies by checking the applicable boxes below:**

None

**Disclosure of Interest:**

None Declared

